# The ability of different peer review procedures to flag problematic publications

**DOI:** 10.1007/s11192-018-2969-2

**Published:** 2018-11-29

**Authors:** S. P. J. M. Horbach, W. Halffman

**Affiliations:** 1 Institute for Science in Society, Faculty of Science, Radboud University, P.O. box 9010, 6500 GL Nijmegen, The Netherlands; 2 Centre for Science and Technology Studies (CWTS), Faculty of Social Sciences, Leiden University, Wassenaarseweg 62A, 2333 AL Leiden, The Netherlands

**Keywords:** Research integrity, Peer review, Retractions, Scientific publishing

## Abstract

There is a mounting worry about erroneous and outright fraudulent research that gets published in the scientific literature. Although peer review’s ability to filter out such publications is contentious, several peer review innovations attempt to do just that. However, there is very little systematic evidence documenting the ability of different review procedures to flag problematic publications. In this article, we use survey data on peer review in a wide range of journals to compare the retraction rates of specific review procedures, using the Retraction Watch database. We were able to identify which peer review procedures were used since 2000 for 361 journals, publishing a total of 833,172 articles, of which 670 were retracted. After addressing the dual character of retractions, signalling both a failure to identify problems prior to publication, but also the willingness to correct mistakes, we empirically assess review procedures. With considerable conceptual caveats, we were able to identify peer review procedures that seem able to detect problematic research better than others. Results were verified for disciplinary differences and variation between reasons for retraction. This leads to informed recommendations for journal editors about strengths and weaknesses of specific peer review procedures, allowing them to select review procedures that address issues most relevant to their field.

**Electronic supplementary material:**

The online version of this article (10.1007/s11192-018-2969-2) contains supplementary material, which is available to authorized users.

## Introduction

There is a growing concern about erroneous or fraudulent research that gets published in the scientific literature. Mainly originating in the biomedical sciences, scholars have demonstrated that a large proportion of published articles contains flaws (Ioannidis 2005), are not reproducible (Open Science Collaboration 2015), involve questionable research practices, or even outright misconduct (e.g. Horbach and Halffman 2017b; Nuijten et al. 2016). The potential consequences of such problematic publications include sending research on unfruitful avenues, wasting valuable research time and funds, skewing meta-analyses or systematic reviews (Tramer et al. 1997), or building policy recommendations and medical treatments on shaky grounds. Others have expressed worries over the potential reputational damage to science (Drenth 2006).

Retractions are one of research journals’ tools to correct the scientific record or redress fraudulent publication credits. The transition to electronic publishing has made it relatively easy to retroactively flag problematic publications. With considerable hesitation over the possible reputational damage for both authors and journals, editors have nonetheless increased the use of this instrument to rectify problematic publications (Cokol et al. 2008; He 2013). As a result, the number of retractions has grown sharply over the last decades, which has led some scholars and journalists to use retractions as a window to study problematic research practices (e.g. Fanelli 2009; Fanelli et al. 2015).

Besides attempts to retroactively redress problematic publications, there have also been several calls and initiatives to try and improve journals’ peer review systems to prevent problematic publications in the first place. However, journals’ use of peer review to identify fraudulent research is highly contentious. Some argue that peer review was never intended to track fraud and cannot be expected to do so (Biagioli 2002; Smith 2006). Nevertheless, concerns about tracing data manipulation, plagiarism, sloppy statistics, inappropriate referencing, or similar improper behaviour have explicitly motivated several recent peer review innovations (e.g. Scheman and Bennett 2017; Epskamp and Nuijten 2014; Kharasch and Houle 2018; Horbach and Halffman 2018). Such initiatives include the use of various software tools, such as text similarity software or ‘plagiarism scanners’ (Zhang 2010), but also modifications in peer review procedures, such as the use of checklists or specialised statistics reviewers (Goodman 2017).

These contradictory expectations raise the question to what extent peer review innovations are able to catch problematic research reports before publication and thereby prevent the need for retractions further down the line. In fact, while various actors have been calling for ‘evidence-based’ improvements of the peer review system, very little is known about the performance of different review models (Rennie 2016). We will address this knowledge-gap in this study. More specifically, in this article we investigate whether and how different peer review procedures (e.g. blind, double blind, or ‘open’) and instruments are related to retraction rates. Using survey data on peer review procedures in a wide range of journals, we relate journal articles to the review procedure they went through. Subsequently, we analyse the relative number of retractions for each review procedure, taking a closer look at the research discipline in which the article was published and, in case of retracted articles, the reason for retraction. Thereby we analyse the effectiveness of different peer review procedures to detect various types of errors and questionable or fraudulent research practices. This leads to informed recommendations for journal editors about strengths and weaknesses of peer review procedures, allowing them to select review procedures that address issues relevant to their field.

In this article, we first discuss the contentious expectations for journal peer review and the motivation behind its recent innovations, resulting in a taxonomy of peer review procedures. Second, we discuss retractions and their ambivalent nature as both indicator *of* problematic research and measure *against* problematic research, leading to important caveats about the interpretation of our findings. Third, we describe the methods used, with a survey among editors and the use of the *Retraction Watch* database. Next, we present and discuss our results per peer review procedure, along with a discussion of the motivation behind them and a discussion of the findings. In the final section, we provide an overview of the statistically significant relations and discuss the limitations of our findings, the consequences and recommendations for journal editors, as well as some questions for further research.

## Theoretical framework

### Diversity and expectations in peer review

Self-regulating mechanisms are considered an important means of ensuring the quality of the published literature (Stroebe et al. 2012; Hiney 2015). Among them, the peer review system holds a central position (Horner and Minifie 2011). Especially after WWII, peer review of publications gradually came to be seen as the best quality guarantee for the research record, spreading from the natural sciences to other disciplines (Cintas 2016; Baldwin 2017; Fyfe et al. 2017).

Even though the expectation that peer review can detect erroneous research has historically been criticised, it is currently expressed with increasing intensity (LaFollette 1992; Stroebe et al. 2012). Mainly editors and publishers have long asserted that peer review was never designed, nor meant to detect errors or fraud in submitted manuscripts. However, various other actors have increasingly come to expect peer review to help assure a fraud-free published literature. This trend mainly emerged as a response to high subscription costs for journals, leading users to demand better quality assurance, as well as to novel technologies and techniques that promise to help editors and journals to detect errors in research (Fyfe et al. 2017; Larivière et al. 2015).

Peer review procedures are highly diverse, with innovations appearing at an increasing pace (Horbach and Halffman 2018). Whereas the use of external reviewers did not become common practice till well after WWII (Baldwin 2015, Baldwin 2017), subsequent innovations in review procedures have emerged quickly. These include changes in the relative timing of review in the publication process (Chambers 2013; Nosek and Lakens 2014; Knoepfler 2015), the range and anonymity of actors involved in the review process and the interaction between them (Pontille and Torny 2014; Okike et al. 2016; Godlee 2002), the level of cooperation and specialisation in review (Barroga 2013; Goodman 2017), and the use of digital tools to assist review.

However, very little is known about the effectiveness of various peer review procedures to detect erroneous or fraudulent research. Several studies suggest that peer review is currently under severe threat and falling below standards. Faulty and even fraudulent research slips through peer review at alarming rates (Stroebe et al. 2012; Bohannon 2013; Lee et al. 2013; van der Heyden et al. 2009; Claxton 2005). The fact that only very few of the widely reported misconduct cases were detected through peer review (Stroebe et al. 2012) also raises questions about its fraud detection potential. However, even though peer review in general seems to fail to detect problematic research, little is known about the relative effectiveness of its different procedures.

To assess the effectiveness of various review procedures, we use the taxonomy presented in Table 1, based on the peer review inventory in (Horbach and Halffman 2018). The peer review procedures are characterised by twelve key attributes, grouped in four dimensions.

**Table 1:** Procedures of peer review categorized by dimension and attribute

Dimension	Attribute	Range
Selection conditions	Timing	a. No review
		b. Pre-submission (including registered reports)
		c. Pre-publication
		d. Post-publication
	Criteria	a. Methodological rigour and correctness
		b. Anticipated impact (either within or outside of science)
		c. Novelty
		d. Fit with journal’s scope
		e. Other
Identities and access	Type of reviewer	a. Editor-in-chief
		b. Editorial committee
		c. External reviewers selected by authors
		d. External reviewers selected by editor(s)
		e. Wider community/readers
		f. Commercial review platforms
	Anonymity of authors	a. Author identities are blinded to editor and reviewer
		b. Author identities are blinded to reviewer but known to editor
		c. Author identities are known to editor and reviewer
	Anonymity of reviewers	a. Anonymous reviewers
		b. Reviewers’ identities are open to the authors
		c. Reviewers’ identities are open to other reviewers
		d. Reviewers’ identities are open to the reader
	Availability of review reports	a. Review reports are accessible to authors and editors
		b. Review reports are accessible to other reviewers
		c. Review reports are accessible to readers of the published manuscript
		d. Review reports are publicly accessible
	Interaction	a. No interaction between authors/reviewers
		b. Interaction amongst reviewers is facilitated
		c. Author’s responses to review reports are communicated to the reviewer
		d. Interaction between authors and reviewers is facilitated
Specialisation in review	Structure	a. Unstructured
		b. Semi-structured: Reviewers are guided by some open questions or are presented several criteria for judgement
		c. Structured: Review occurs through mandatory forms or checklists to be filled out by reviewers
	Statistical review	a. Not applicable
		b. No special attention is given to statistical review
		c. Incorporated in review
		d. Performed by additional, specialist reviewer
		e. Performed through automatic computer software
	External sources	a. No reviews from external sources are used
		b. Reviews from other (partner) journals accompanying manuscripts rejected elsewhere are used
		c. Reviews from commercial review platforms are used
		d. Reviews performed by the wider community (i.e. not by invited or targeted reviewers) are used
Technological tools in review	Technical support	a. No digital tools are used
		b. Plagiarism detection software is used
		c. Digital tools to assess validity or consistency of statistics are used
		d. Digital tools to detect image manipulation are used
		e. Digital tools to check references are used
		f. Other technical support (e.g. machine learning techniques to assess consistency and completeness)
	Reader commentary	a. No reader commentary facilitated
		b. In-channel reader commentary facilitated
		c. Out-of-channel reader commentary facilitated

### What are retractions?

Retractions are a measure taken by journal editors to remove publications from the official published scientific record (even though the original text remains available, marked as ‘retracted’). A request for retraction may be made by publishers, (co-)authors, research organisations, funders, or any other actor in the research process, but the decision to retract remains an editorial one. Retractions occur for a wide variety of reasons, ranging from honest error to severe cases of research fraud (Hesselmann et al. 2017; Fang et al. 2012).

Retractions are announced through published retraction notices, with a reference to the original publication, and normally also through a warning on the electronic version of the publication. Retraction notices are indexed in databases such as Web of Science or PubMed, but their unstandardized format makes them hard to collect systematically (Hesselmann et al. 2017; Schmidt 2018; Van Leeuwen et al. 2017). In fact, the explanation offered in retraction notices is often obtuse and cryptic. Since 2010, the NGO *Retraction Watch* has been documenting retractions, originally with a journalistic interest in specific misconduct cases. More recently, *Retraction Watch* has developed an online database that provides the most complete overview of retractions currently available, going back to the 20th century (Retraction Watch 2018).

Retractions are a relatively new phenomenon for many research fields. Although the oldest known retraction dates from 1927, retractions occurred only sporadically before 2000 (He 2013). The more traditional response would have been to publish another article, in the form of a rebuttal or challenge to the original publication. The practice became more meaningful as electronic publishing made it possible to add a warning to the original publication, alerting readers accessing the publication online to its problems. Theoretically, this makes it possible to recognise problematic publications without having to know the complete subsequent literature on the matter.

The effects of retractions are complex. Retractions are generally considered a serious reprimand and have detectable negative effects on careers (Azoulay et al. 2017). Some editors may also fear retractions will put a blemish on their journal’s reputation, as retractions could be perceived as sign of a failing editorial policy. In contrast, retractions’ effect on removal of error or fraud from the scientific record is more modest than expected, as many retracted articles continue to be cited, both by their authors and by others (Van Noorden 2011; Madlock-Brown and Eichmann 2015).

In research on scientific integrity, retractions are sometimes used as indicators of misconduct or questionable research practices (Fanelli et al. 2015; Karabag and Berggren 2016; Hesselmann et al. 2017; Montgomery and Oliver 2017). However, this requires extreme caution. A quarter of retractions do not involve misconduct, but honest error (Fang et al. 2012), sometimes even by journals or publishers. Retractions may signal an offence, but also the social reaction to the offence: an editor has decided (or was pressured) to take the relatively severe measure of retraction to repair the scientific record. Retraction rates are therefore both an indicator of a research community having a problem, but also of this research community taking action to redress this problem. Retractions signal trouble, but also the awareness and resolve to address trouble. Inversely, the absence of retractions does not necessarily signal unproblematic research, but might also indicate an unwillingness to act against it.

In this respect, retractions as indicators of misconduct or problematic research suffer from the same problem as crime statistics: crime rates registered by police or the justice system indicate crime as well as crime fighting (Biderman and Reiss 1967). In criminology, this is known as the ‘dark number’ problem (Skogan 1977). The issue with dark numbers in estimating misconduct rates have lead scientists, in analogy with criminologists (Van Buggenhout and Christiaens 2016), to adopt various other ways of collecting data on misconduct and errors in research, including (self-reported) misconduct surveys (Martinson et al. 2005), sometimes using incentives for truth-telling (John et al. 2012); or digital tools for detecting problematic research (Horbach and Halffman 2017a), in addition to retraction rates. The latter arguably suffers most from the dark number problem: retraction rates are lower than other misconduct indicators.

A further complication is that measures to trace misconduct may also define or articulate particular behaviour *as* misconduct. Misconduct and scientific error are not just pre-existing, objectively defined phenomena, but may be re-categorised as such by punitive social reaction (Martin 1992). This is an inherently social process in which power structures play a major role (Martin 1992; Callahan 2017). For example, plagiarism scanners have made it possible to systematically trace text recycling, which has raised awareness and also spurred codification of which forms of text recycling are and are not to be considered acceptable (KNAW 2014). Similarly, statistics scanners may also redefine practices that were common and considered normal in particular research fields as problematic or even fraudulent.

These complications imply that retraction rates are ambivalent indicators of integrity problems and have to be interpreted with caution. Nevertheless, we will show that some interpretable and statistically significant relations can be found between some peer review procedures and retractions, including specific effects such as the prevention of plagiarism, or the effect of post-publication reader commentary.

## Methods

### Data collection

#### Retracted journal articles

**Fig. 1:**
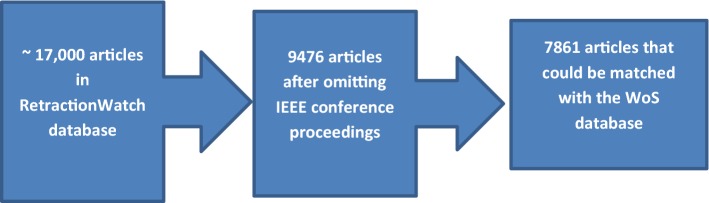
Overview of the retractions data. Starting with the RetractionWatch database, we omitted a large number of retracted conference proceedings by IEEE. The final set consists of the resulting retracted articles that are contained in the WoS database

Retracted publications were gathered from the *Retraction Watch* online database (Retraction Watch 2018) on December 11th 2017, at which point it contained 9476 retractions for journal papers (omitting roughly 7500 retracted conference proceedings from the Institute for Electrical and Electronics Engineering [IEEE], which were irrelevant for our focus on journal review). We collected information on the title of the retracted article, the Digital Object Identifier (doi), the Pubmed ID, and the reason for retraction. Using the doi and Pubmed ID, the records were subsequently matched with the Web of Science (WoS) database. This allowed us to gather additional data on the retracted articles available in WoS, such as date of publication, publishing journal, research discipline, and citations to the retracted article. This yielded a list of 7861 retractions. Figure 1 displays an overview of the selected retractions.

#### Peer review procedure questionnaire

Unfortunately, journal web pages present surprisingly incomplete information about their peer review procedures, even for procedures currently in use. This information had to be gathered through a short questionnaire among journal editors. The questionnaire consisted of 12 questions, each on a specific attribute of the journal’s peer review (Table 1). The questionnaire can be found in the supplementary material. In addition, respondents were asked to indicate whether, when and how any of these attributes was modified since 2000. Hence the questionnaire allowed us to identify precisely which peer review procedure was used by a specific journal in any specific year since 2000. Subsequently, this allowed us to match every published paper to the specific peer review procedure it went through. Although very simple, we nevertheless pre-tested the questionnaire with two editors to avoid ambiguities, leading to minor modifications.

#### Mailing

Journal editors were contacted via email. Email addresses were gathered in two ways. First, we sampled all 2017 articles indexed in WoS as ‘editorial material’ and extracted the email address of the corresponding author, on the assumption that authors of ‘editorial material’ would very likely be editors. This yielded a list of 58,763 unique email addresses, covering a total of 6245 different journals. We subsequently collected one random email address for each of these journals. Second, because of our specific interest in journals with a substantive amount of retractions, we also manually collected editorial email addresses of journals with at least 10 retractions, in case they had been omitted via our first sampling strategy. Combined, this yielded editorial email addresses for a total of 6331 journals.

The questionnaire was initially sent on February 23rd 2018 and reminders were sent on March 12th 2018 and March 19th 2018. For the second reminder, we used alternative editorial email addresses from these journals, if available in our database. We received 326 automatic response messages of emails that could, due to various reasons, not be delivered. In addition, 113 people responded that they were not able to fill in the questionnaire, for example because they were not or no longer an editor. Hence, a total of 5892 (= 6331-326-113) journals were effectively reached. After sending out the questionnaire, several respondents offered to further disseminate the questionnaire among their networks, which we gratefully accepted. Hence the questionnaire was subsequently distributed among the European Association of Science Editors (EASE), the International Society of Managing and Technical Editors (ISMTE) and through the newsletter of the Committee on Publication Ethics (COPE). We stopped collecting responses on April 5th 2018.

After the reminders, we eventually obtained a total of 361 useful responses. The final response rate of 6.12% is low, but comparable to, or even higher, than response rates of similar online surveys among journal editors or authors regarding issues related to academic integrity (Hopp and Hoover 2017; Stitzel et al. 2018). Nevertheless, our sample covers a wide range of research fields and reflects the distribution of journals over research fields.

### Data analysis

Using the database of retracted articles and the Web of Science database of published articles, we identified the number of published and the number of retracted articles per journal per year. We limited the analysis to publications indexed in WoS as research articles, rather than, for instance, editorials or book reviews. In total, the journals responding to our survey published 833,172 articles since 2000, of which 670 were retracted. This constitutes the eventual sample. Each article serves as a record in our dataset, thereby taking individual articles, rather than journals, as our unit of analysis. Taking journals as unit of analysis would create considerable complications. First, this would imply using retraction rates of single journals as our measure, but the numbers of publications vary substantially between journals. Hence retraction rates are of unequal accuracy. Second, as journals changed their review procedures since 2000, such as with the introduction of plagiarism scanners, a journal is not a constant unit of analysis. Because we asked editors when and how their journal’s peer review procedures changed, we know what procedure articles went through based on their publication date, assuming editors report procedures accurately and procedures are applied consistently. As this may not always be the case, we acknowledge that we actually test the relation between peer review *procedures* and retractions, rather than peer review *practices* and retractions: the actual review may differ from the formal procedure.

We attributed to each record: the peer review procedure that the article went through, for each of the 12 attributes identified in the survey; the research area that the article belongs to (Social Science and Humanities, Biomedical and Health Science, Physical Sciences and Engineering, Life and Earth Science, or Mathematics and Computer Sciences), based on the classification in research areas of the Leiden Ranking (Waltman and van Eck 2012; CWTS 2018); and, in case the article was retracted, the reason for retraction, based on the data from *Retraction Watch*. Table 2 presents an overview of the article distribution over research areas. The retractions in our sample reflect the distribution of articles and retractions over fields. The high number of biomedical retractions corresponds to the more frequent use of retractions in this field. Note that some journals responding to our survey are not indexed by the Leiden Ranking and hence are not classified in a research field, causing the numbers per research area to add up to (slightly) less than the total number of articles or retractions.

**Table 2:** The number of articles and retracted articles since 2000 in our sample, according to research area, as defined in the Leiden Ranking

Research area	Social science and humanities	Biomedical and health sciences	Physical sciences and engineering	Life and earth sciences	Mathematics and computer sciences	Total
Total articles published	1,660,394	6,325,415	5,717,615	2,948,177	1,605,903	18,257,504
Articles in our sample	53,922	382,950	196,845	160,126	38,225	833,172
% of total articles in our sample	3.25	6.05	3.44	5.43	2.38	4.56
Total retractions	380	4394	1693	800	281	7547
Retractions in our sample	30	368	183	67	21	670
% of retracted articles in our sample	7.90	8.38	10.81	8.38	7.48	8.86

**Fig. 2:**
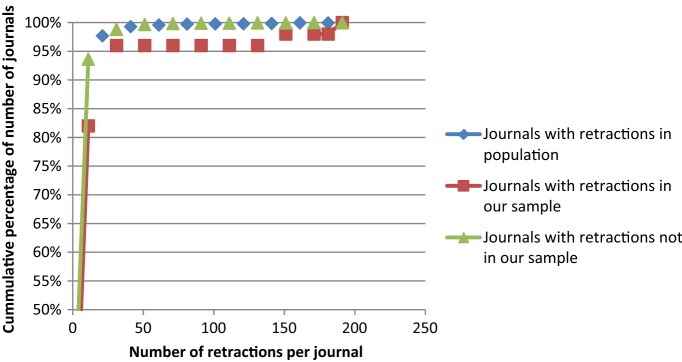
Cumulative distribution of retractions over journals, either in the entire population, in our sample, or outside of our sample

Table 2 shows that our sample contains a relatively high share of retractions. Indeed, relatively many journals with a high number of retractions have responded to our survey. The same observation can be made from figure, showing the number of journals with a specific number of retractions. It shows that our sample contains a good representation of both journals with large and small numbers of retractions, but with a slight over-representation of journals with large numbers of retractions. For example, Fig. 2 demonstrates that while in general 90% of all journals has fewer than 8 retractions, in our sample this is only 70% of all journals. In contrast, while in the general population of journals the top 5% of journals with most retractions has at least 12 retractions, the top 5% in our sample only starts at 24 retractions.

We suspect that editors with previous experience with retractions were more likely to answer the survey, but we also note that our sampling strategy included more journals with a relatively high number of retractions among the recipients of our survey.

The data from *Retraction Watch* was classified in nine categories of reasons for retraction: Plagiarism/Duplication of text, Falsification/Fabrication, Ethical concerns, Authorship issues, Issues with References, Image manipulation/Data issues, (Honest) Errors, Fake Review, and Misconduct (grouped). Misconduct (grouped) is a category encompassing all other categories except (honest) error and further includes the general labels referring to misconduct as given by *Retraction Watch*. A complete classification of the reasons for retraction as presented by *Retraction Watch* into the categories specified above, can be found in the supplementary material. Note that a single retraction usually occurs due to various reasons, classifying individual retractions in multiple categories. In addition we note that *Retraction Watch* also uses the ‘error’ category in cases where other reasons for retraction, such as misconduct, cannot be proven (even though strong suggestion to the contrary might exist).

For each of the twelve characteristics of peer review we studied, we analysed whether the review procedure had a (significant) association with the chance of a paper being retracted. Significance was tested using a log likelihood ratio-test on the ratios of retracted and non-retracted papers. In addition, subgroup-specific effects for the five research areas were tested using binary logistic regression as interaction test between the peer review procedure and the research discipline. Here we use the Wald-statistic on the logistic model with the interaction term, compared to the model without the interaction term. Last, specific effects for the various retraction reasons were analysed, using an ANOVA test to analyse differences in mean retraction numbers, and multinomial logistic regression to analyse the effect size of various peer review procedures on retraction rates. Analyses were performed using the SPSS 25.0 statistics software.

## Results

This section presents the results of our analyses, grouped by the various attributes of peer review presented in Table 1. For each of the twelve attributes included in our survey, we briefly outline some of the differences between the various procedures, the original rationale behind their development and some effects on rates of problematic publications or retractions expected in the literature.^1^ We should note that many of these procedures may be combined in the review process of journals, for instance using both registered reports as well as pre-publication review, or involving multiple actors in the review process. Subsequently, we present the results of our analyses according to whether and how these peer review procedures are associated with difference in the rate of retraction, whether research area is a (significantly) mediating factor and whether differences in retraction rate for different reasons of retraction are visible. We conclude with a short discussion of the results.

### Timing

Traditionally, peer review occurs between the submission and publication of a manuscript. However, over the past decades, new peer review procedures have been proposed for different phases of the publication process. Most notably these include: pre-submission review (e.g. through registered reports) (Chambers 2013; Nosek and Lakens 2014; Mellor 2016), in which articles are reviewed prior to data collection based on their rationale, research question and proposed method; and post-publication review (Knoepfler 2015; Pöschl 2012), in which articles are reviewed only after publication, potentially involving a wider community rather than merely invited reviewers. The latter procedure was mainly introduced in order to speed up publication and enhance fast knowledge exchange, whereas the pre-submission procedure was primarily introduced to foster publication of negative or null-results and deter researchers from hunting for spectacular outcomes (Chambers et al. 2014; Nosek and Lakens 2014).

Our results suggest that the pre-submission system is indeed related to fewer retracted articles (Table 3): in total, 7.6% of all articles went through pre-submission review, whereas only 4.8% of retractions went through this review procedure (*Λ*(3) = 18.899, *p* < 0.001). Due to the ambivalent nature of retractions as both indicator of undetected errors or of the willingness to repair errors, this could mean that these journals are less prone to take action after publication. However, since this system is used with the explicit intention to prevent tweaking of data or statistics, it seems highly unlikely that this lower retraction rate is due to lax editorial attitudes towards problematic research.

**Table 3:** Timing of peer review relative to the publication process related to number of non-retracted and retracted articles in our sample

At what stage of the publication process does review take place?	Retracted	95% CI	Non-retracted	95% CI
No review takes place	0 (0.0%)	0.0–0.0	648 (0.1%)	0.1–0.1
Pre-submission review (including registered reports)	32 (4.8%)	3.2–6.4	63,262 (7.6%)	7.5–7.7
Pre-publication review	645 (96.3%)	94.8–97.7	812,362 (97.6%)	97.5–97.6
Post-publication review	0 (0.0%)	0.0–0.0	7008 (0.8%)	0.8–0.9

The rates for traditional pre-publication review (97.6% vs. 96.3%) and post-publication review (0.8% vs. 0.0%) did not show significant differences. However, the fact that no retractions were reported in journals using post-publication review is interesting. It might suggest that potential issues are dealt with in review and commentaries, rather than using retractions as a mechanism to correct the literature, but the number of publications reviewed in this way is still relatively low. No significant interactions were found with respect to research area (WALD = 5.445, *df* = 5, *p* = 0.364), nor reasons for retraction (*F*(1,1266) = 5.409, *p* = 0.020).

### Review criteria

Journals use a variety of review criteria. Commonly, methodological rigour and correctness, conceptual soundness, and fit with the journal’s scope are used as assessment criteria. However, also scientific novelty and anticipated impact (either within or outside of science) are used to assess manuscripts. Some journals have deliberately decided *not* to take factors like novelty nor anticipated impact into account when judging manuscripts (BMJ Open 2018; PLOS 2018; Sage Open 2018). Their rationale for doing so is to allow all valid research (i.e. methodologically and conceptually sound research) to be published, irrespective of whether results are positive or negative, and irrespective of novelty or impact. Thereby they facilitate the publication of replication studies and do not incentivise authors to obtain spectacular, new or (significantly) positive results. This arguably takes away incentives for questionable research practices and may hence foster research integrity.

The results of our analysis (Table 4) suggest that journals taking novelty and anticipated impact into account when assessing manuscripts are indeed associated with more retractions. The criteria used for assessing articles demonstrate a significant impact on the number of retractions (*Λ*(3) = 18.779, *p* < 0.001), with significantly more retractions for journals using novelty and anticipated impact as assessment criteria. No significant interactions were observed regarding research area (WALD = 16.171, *df* = 12, *p* = 0.161), nor reason for retraction (*F*(3,4665) = 1.220, *p* = 0.301), suggesting that the effect is homogeneous with respect to research discipline and type of problematic research.

**Table 4:** Review criteria related to number of non-retracted and retracted articles in our sample

What quality criteria does your journal use for peer review?	Retracted	95% CI	Non-retracted	95% CI
Methodological rigour and correctness	666 (99.4%)	99.1–99.7	810,932 (97.4%)	97.4–97.4
Anticipated impact (either within or outside of science)	549 (81.9%)	80.4–83.5	523,629 (62.9%)	62.8–63.0
Novelty	610 (91.0%)	89.9–92.2	697,846 (83.8%)	83.8–83.9
Fit with journal’s scope	641 (95.7%)	94.9–96.5	733,670 (88.1%)	88.1–88.2

The higher retraction levels among journals aiming to publish highly relevant and novel research, usually journals with high impact factors, has also been established in previous research on retractions (Steen 2011; He 2013). As such, focussing on high-impact and novel research might be a deliberate high-risk/high-gain strategy for journals, potentially leading to high impact factors and citation scores, but also to a higher risk of having to retract articles. Here too, the lower retraction rate seems more plausibly associated with prevention of problematic publications, rather than with the willingness to rectify it. In fact, journals that use anticipated impact as a selection criterion have a significantly higher rejection rate (70% vs 63%, t(280) = − 3.043, *p* = 0.016). Apparently, they have ‘more to choose from’ than journals that do not use impact as criterion, and/or have a tighter limit on the number of articles they can publish (e.g. printed versus exclusively electronic journals). However, the higher retraction rates suggest that these journals either attract more problematic submissions or are less capable of filtering them.

In addition, it could be expected that the strategy to select articles with the highest anticipated impact would be expressed in a higher journal impact factor (JIF). However, the journals that use anticipated impact as a selection criterion, on average, do not have a higher journal impact factor. On the contrary, journals in our sample using impact as a selection criterion have a slightly *lower* 2016 JIF than those which do not (2,51 vs. 2,86). The precise relation between impact as a selection criterion, JIF, and retraction rates would have to be analysed in a larger, multivariate analysis, but our findings suggest the impact criterion provokes more retractions *and* fails to increase the JIF rating.

### Type of reviewers

The use of external reviewers, i.e. researchers not directly affiliated with the journal, did not become standard practice until well after WWII (Baldwin 2017). Still today, the actors performing reviews range from the editor-in-chief, editorial committee members, external reviewers (either suggested by authors or merely selected by editors), to the wider community (usually in post-publication review), or even independent commercial review platforms (Research Square 2017; Tennant et al. 2017). The latter have recently emerged as organisations to which authors may submit their manuscript for review, after which the manuscript together with review reports (or certain assigned ‘badges’) are sent to a suitable journal (PubPeer Foundation 2017; Research Square 2017). This has mainly been introduced to prevent manuscripts from going through several rounds of review after rejection at an initial journal, thereby decreasing the burden on the peer review system.

Our analysis shows a significant impact of the actor type performing the review (*Λ*(5) = 116.527, *p* < 0.0001), with relatively few retractions occurring when editors-in-chief or the wider community are involved in review (Table 5). In addition, a significant difference was found regarding the reason for retraction (*F*(4,2782) = 10.538, *p* < 0.001): when the editor-in-chief, the editorial committee or author-suggested reviewers are involved, relatively few retractions appear for fake review reports or issues with references, while relatively more retractions occur for authorship or ethical issues. Finding relatively few retractions for fake peer review when author-suggested reviewers are used, is somewhat puzzling, as these types of actors seem most vulnerable to fraud with review reports. More research will be needed to elucidate the mechanism underlying this association.

**Table 5:** Identity of reviewer related to number of non-retracted and retracted articles in our sample

Type of reviewers	Retracted	95% CI	Non-retracted	95% CI
Editor-in-chief	197 (29.4%)	27.2–31.6	347,035 (41.7%)	41.6–41.8
Editorial committee	376 (56.1%)	53.7–58.5	439,754 (52.8%)	52.8–52.9
External reviewers suggested by authors	372 (55.5%)	53.1–57.9	445,636 (53.5%)	53.5–53.6
External reviewers suggested and selected by editor(s)	653 (97.5%)	96.7–98.2	805,787 (96.8%)	96.8–96.8
Wider community/readers	34 (5.1%)	4.0–6.1	146,502 (17.6%)	17.5–17.6
Commercial review platforms	0 (0.0%)	0.0–0.0	9192 (1.1%)	1.1–1.1

The finding that involvement of the wider community is related to fewer retractions is in line with expectations expressed in the literature, which suggest that wider involvement would lead to higher levels of scrutiny and hence a higher quality review, as well as a stronger deterring effect diverting fraudulent papers away from these journals (Harris et al. 2015). Our result that involvement of the editor-in-chief would lead to higher quality review also raises some further questions. Future research could look at this in more detail, for instance specifying the role of the editor-in-chief in the review process or distinguishing between editors for whom editorial work is their main occupation and those doing it more or less voluntarily in their free time. In any case, again, involvement of such actors seems unlikely to be related to poor willingness to address problematic research. Hence, in this case also, low retraction rates are more likely explained by more effectiveness to detect such research in an early phase.

### Author anonymity

In the early days of peer review, editors and reviewers were (nearly) always aware of authors’ identities, whereas authors knew the identity of the editor-in-chief, but not necessarily of the editorial committee or invited outside reviewers (single-blind review). Responding to issues of equality and fairness (Zuckerman and Merton 1971; Peters and Ceci 1982), the systems of double-blind and triple-blind review were introduced, in which author identities were blinded to reviewers and editors respectively (Pontille and Torny 2014). The ambition of these innovations was to judge manuscripts on content rather than extraneous factors such as authors’ gender, affiliation or nationality.

We analysed the impact of blinding author identities to editors and/or reviewers (Table 6). The results demonstrate a significantly lower rate of retractions in case author identities are blinded to the reviewer (*Λ*(2) = 106.042, *p* < 0.0001). The effect can be witnessed in all research areas, but is especially strong in the social sciences and humanities. In this research area, 79% of all articles went through double-blind review, whereas only 13% of all retracted articles went through this review procedure. In contrast, only 19% of the articles were reviewed in a procedure allowing reviewers to see authors’ identities, whereas 87% of all retractions went through such review. The figures for biomedical and health sciences show a similar, but weaker relation (83% of articles did not have author identities blinded during review, but 95% of retractions occur in this procedure). For the other research areas similar trends were found, but no significant differences occurred. In addition, significant differences occurred when comparing the various reasons for retraction (*F*(1,1260) = 10.630, *p* = 0.001), with the strongest effects for the category of retractions due to fake review, ethical violations, and misconduct.

**Table 6:** Level of author anonymity during peer review related to the number of non-retracted and retracted articles per research area

Level of author anonymity		Author identities are known to editor and reviewer	Author identities are blinded to reviewer but known to editor	Author identities are blinded to editor and reviewer
All articles (percentage of all articles)		692,920 (83.2%)	135,019 (16.2%)	7011 (0.8%)
Non-retracted (percentage of non-retracted papers)	Total	692,280 (83.2%)	134,989 (16.2%)	7011 (0.8%)
	Social Science & Humanities	10,206 (18.9%)	42,819 (79.5%)	1192 (2.2%)
	Biomedical and Health Sciences	317,182 (82.9%)	65,329 (17.1%)	1327 (0.3%)
	Physical Sciences & Engineering	181,201 (92.1%)	12,000 (6.1%)	3461 (1.8%)
	Life and Earth Sciences	146,040 (91.2%)	13,186 (8.2%)	1032 (0.6%)
	Mathematics & Computer Sciences	36,849 (96.5%)	1355 (3.5%)	0 (0.0%)
Retracted (percentage of retracted papers)	Total	640 (95.5%)	30 (4.5%)	0 (0.0%)
	Social Science & Humanities	26 (86.7%)	4 (13.3%)	0 (0.0%)
	Biomedical & Health Sciences	348 (94.6%)	20 (5.4%)	0 (0.0%)
	Physical Sciences & Engineering	180 (98.4%)	3 (1.6%)	0 (0.0%)
	Life and Earth Sciences	65 (97.0%)	2 (3.0%)	0 (0.0%)
	Mathematics and Computer Sciences	20 (95.4%)	1 (4.8%)	0 (0.0%)

Studies in psychology and economics have previously suggested that people are more strict when reviewing or judging the unknown rather than the known or the familiar (Cao et al. 2009). Our results suggest the same to be true in academic peer review. In addition, one could argue that, especially in Social Sciences and Humanities, adopting a single-blind review format is a sign of innovation and commitment to act on problematic research. Hence the higher retraction rates might here indicate a higher willingness to address issues, rather than a poorer capability to detect them.

However, what specific mechanism accounts for the difference in retraction rate between single- and double-blind reviewed papers remains to be studied. This is especially so regarding the current discussion about the effectiveness of blinding in the digital age, in which authors are easily identified with a simple Google-search.

### Reviewer anonymity

Similar to the anonymity of the author, some discussions regarding peer review procedures have centred around the anonymity of the reviewer (Amsen 2014; Ross-Hellauer 2017). Contrary to the system of double- or triple-blind review, open review has been proposed as a way to tackle reviewer bias by rendering the review process more transparent (Smith 1999; Godlee 2002). The expectation is that by disclosing the identity of the reviewer to either the authors of the submitted manuscript, other reviewers of the same manuscript, the readers of the published manuscript, or even the general public, reviewers are held accountable for their choices while they do receive credit for their work. The combination of both incentives is argued to facilitate more rigorous review, thereby augmenting the likelihood of detecting erroneous or fraudulent research (Walker and Rocha da Silva 2015; Ross-Hellauer 2017).

Our data (Table 7) does not seem to uphold the claim that known reviewer identities increase the likelihood of retracted papers (*Λ*(3) = 5.964, *p* = 0.0494). Neither do we find significant differences when correcting for research fields (WALD = 15.717, *df* = 7, *p* = 0.028) nor reasons for retraction (*F*(3,1262) = 2.839, *p* = 0.784). This might mainly be due to the fact that an overwhelming majority of the articles, as well as the retractions, goes through the same review procedure: a system in which reviewer identities are blinded to all relevant actors. Hence, to properly study the influence of this review attribute other research strategies such as randomised trails or other intervention studies could be employed.

**Table 7:** Level of reviewer anonymity related to number of non-retracted and retracted articles in our sample

Extent to which reviewers are anonymised	Retracted	95% CI	Non-retracted	95% CI
Reviewers are anonymous (both to authors and other reviewers as well as to readers of the published manuscript)	654 (97.6%)	96.5–98.8	816,784 (98.1%)	98.1–98.1
Reviewer identities are known to other reviewers of the same manuscript	6 (0.9%)	0.2–1.6	4004 (0.5%)	0.5–0.5
Reviewer identities are known to the authors	9 (1.3%)	0.5–2.2	5412 (0.7%)	0.6–0.7
Reviewer identities are known to the readers of the published manuscript	5 (0.7%)	0.1–1.4	4782 (0.6%)	0.6–0.6

### Review reports

In addition to disclosing reviewer identities, open review frameworks have proposed to also make the review reports accessible. We distinguish four levels of accessibility: review reports accessible (1) to authors and editors, (2) to other reviewers of the same manuscript, (3) to readers of the published manuscript, and (4) to the wider public, i.e. without restrictions (Walker and Rocha da Silva 2015; Ross-Hellauer 2017). Making review reports widely accessible has been proposed with the same rationale as disclosing reviewer identities: it provides a transparent and hence supposedly more thorough review process.

In our data (Table 8) we found no significant influence of the accessibility of review reports on the number of retractions (*Λ*(3) = 9.081, *p* = 0.0128). However, we did find some specific influences when regarding research area (WALD = 47.551, *df* = 5, *p* < 0.0001) and the reason for retraction (*F*(3,1821) = 6.897, *p* < 0.001). Making review reports accessible not only to authors and editors, but also to other reviewers of the same manuscript was associated with fewer retraction due to fake reviews and issues with references, while in this case we see an increase in the rate of retractions due to plagiarism, falsification, image and/or data issues and ethical violations. The fact that no significant effects were measured for the other two review procedures, those in which reports are shared with the manuscript’s readers or the wider public, might again be due to the low number of articles and retractions going through these review procedures. Again, other research set-ups could be employed to study the effect of making review reports more or less widely accessible on the quality of review.

**Table 8:** Accessibility of review reports related to number of non-retracted and retracted articles in our sample

Accessibility of review reports	Retracted	95% CI	Non-retracted	95% CI
Review reports are accessible to authors and editors	619 (92.4%)	90.8–94.0	811,087 (97.4%)	97.4–97.5
Review reports are accessible to other reviewers	448 (66.9%)	64.1–69.7	487,933 (58.6%)	58.5–58.7
Review reports are accessible to readers of the published manuscript	5 (0.7%)	0.2–1.3	4790 (0.6%)	0.6–0.6
Review reports are publicly accessible	2 (0.3%)	0.0–0.6	2108 (0.3%)	0.2–0.3

### Interaction between actors

Besides sharing review reports or disclosing identities, some journals have introduced review procedures in which interaction between various actors in the review process is facilitated. This includes modest levels of interaction by allowing reviewers to read author responses to their review report, but also goes further by facilitating interaction between reviewers of the same manuscript (Schekman et al. 2013; EMBO Press 2017), or even facilitating direct communication between authors and reviewers of a manuscript (on top of formal communication by means of review reports and responses to them) (Amsen 2014; Frontiers 2014). Again, a quest for transparency and accountability in review were the main motivators for introducing these review procedures. In addition, they are claimed to improve the quality of reviews by allowing actors to discuss and respond efficiently to reviewers’ questions or comments.

The data from our study (Table 9) actually rather suggest that the opposite is true, finding significantly fewer retractions when no interaction between authors and reviewers is facilitated and relatively more retractions when authors are allowed to respond to review reports (*Λ*(3) = 126.4, *p* < 0.0001). More specifically, allowing no interaction reduces the likelihood of retractions for fake review, ethical issues and misconduct in general. Contrarily, allowing authors to respond to review reports increases the likelihood of retractions occurring for fake review, ethical concerns or issues with references (*F*(3,1405) = 21.269, *p* < 0.001). Research area was also found to be a significantly mediating factor (WALD = 85.710, *df* = 12, *p* < 0.0001) with stronger effects in the biomedical and health sciences as well as the physical sciences and engineering.

**Table 9:** Level of interaction between authors and reviewers related to number of non-retracted and retracted articles in our sample

Interaction between authors and reviewers	Retracted	95% CI	Non-retracted	95% CI
No interaction between authors or reviewers is facilitated	130 (19.4%)	16.6–22.2	339,782 (40.8%)	40.7–40.9
Author’s responses to review reports are communicated to the reviewer	562 (83.9%)	81.3–86.5	573,308 (68.9%)	68.8–69.0
Interaction between reviewers is facilitated	48 (9.0%)	5.3–9.0	75,100 (9.0%)	9.0–9.1
Interaction between authors and reviewers is facilitated (on top of formal review reports and formal responses to review reports)	18 (2.7%)	1.5–3.8	52,088 (6.3%)	6.2–6.3

In particular, it might be deemed surprising that interaction between reviewers is not associated with lower retraction rates, as more interaction is expected to lead to higher scrutiny during review and hence to fewer retractions. Indeed, in other settings, such as detecting medication errors, it has been suggested that higher levels of cooperation and interaction would be beneficial for effective error detection (Kaushal et al. 2001). Similar relations might be expected from editorial peer review. The specific effect (or lack thereof) of interaction and communication between reviewers is open to future research.

### Checklists: level of structure in review criteria

Another salient difference distinguishing review procedures is the level of structure that editors require from their reviewers. We distinguish three levels of structure: structured, when reviewers are asked to fill out a form or checklist listing specific (closed) questions or to rate specific aspects of the manuscript; semi-structured, when reviewers are presented a list of guiding questions or criteria that might assist them in writing their review; and unstructured, when reviewers receive a manuscript for review without further guidance about review criteria.

Our data suggests (Table 10) that the level of structure plays a significant role in the relative number of retractions appearing after peer review (*Λ*(2) = 58.907, *p* < 0.0001), with fewer retractions appearing in either structured and unstructured review, but more retractions appearing after semi-structured review. Specifically, semi-structured review is related to significantly more retractions for fake review, authorship and ethical issues and concerns over references (*F*(2,1382) = 12.538, *p* < 0.001). In addition, subject area turned out to be a significant mediating factor (WALD = 145.578, *df* = 8, *p* < 0.0001), with particularly strong effects in Social Science and Humanities and Mathematics and Computer Science, and relatively weak effects in Life and Earth Sciences.

**Table 10:** Level of structure in review related to the number of non-retracted and retracted articles per research area

Level of structure		Unstructured	Semi-structured	Structured
All articles (percentage of all articles)		126,282 (15.2%)	627,559 (75.3%)	158,274 (19.0%)
Non-retracted (percentage of non-retracted papers)	Total	126,233 (15.2%)	626,971 (75.3%)	158,184 (19.0%)
	Social Science & Humanities	17,275 (32.1%)	33,574 (62.3%)	14,652 (27.2%)
	Biomedical & Health Sciences	45,414 (11.9%)	270,776 (70.8%)	82,097 (21.5%)
	Physical Sciences & Engineering	19,946 (10.1%)	171,251 (87.1%)	51,172 (26.0%)
	Life & Earth Sciences	35,010 (21.9%)	122,726 (76.7%)	7783 (4.9%)
	Mathematics & Computer Sciences	8290 (21.7%)	27,910 (73.1%)	2113 (5.5%)
Retracted (percentage of retracted papers)	Total	49 (7.3%)	588 (87.8%)	90 (13.4%)
	Social Science & Humanities	1 (3.3%)	26 (86.7%)	3 (10.0%)
	Biomedical & Health sciences	21 (5.7%)	303 (82.3%)	47 (12.8%)
	Physical Sciences & Engineering	1 (0.5%)	182 (99.5%)	10 (5.5%)
	Life & Earth Sciences	26 (38.8%)	56 (83.6%)	29 (43.3%)
	Mathematics & Computer Sciences	0 (0.0%)	21 (100.0%)	0 (0.0%)

Interestingly, both extremes of the spectrum appear related to fewest retractions. This suggests that either guiding reviewers very specifically through the review process or leaving them to decide on appropriate ways of reviewing themselves is most effective in detecting problematic publications. Alternatively, partly guiding reviewers seems to be least effective. We could speculate that reviewers in this case would only consider those aspects referred to in their checklist, while editors might expect them to take more aspects of the manuscript into account. However, other mechanisms might also be at play. To obtain a better understanding of this phenomenon, future research could compare specific guidelines for reviewers with the retraction rates on a more qualitative level.

Since especially highly structured review procedures were introduced expressly with the intent to address problematic research, it seems improbable that lower retraction rates are to be seen as an indication of unwillingness to address problematic research. However, low retraction rates for the other side of the spectrum, i.e. unstructured review criteria, are harder to interpret in this way.

### Statistics review

Statistical analyses are increasingly recognised as a source of error, questionable research practices, or outright fraud in quantitative scientific papers (Altman 1998; Goodman 2017; Carlisle 2017). Hence, statistics has come under close scrutiny in some journals’ review process. This led several journals to assign specialist statistical reviewers to their review pool already in the 1980s (George 1985). In addition, more recently, several digital tools were developed to assist in the review of statistical analyses (Bakker and Wicherts 2011; Nuijten et al. 2016). These all aim to increase the detection likelihood of statistical errors and misrepresentations.

Our data indeed show (Table 11) a significant influence of how statistics is included in the review process (*Λ*(4) = 138.858, *p* < 0.0001). However, the results do not provide evidence for the effectiveness of assigning specialist statics reviewers or employing digital tools to assist in statistical review. Specifically, we witness more retractions appearing in journals that state that statistics is not relevant for their journal, while less retractions appear in journals either paying ‘no special attention’ to review, incorporate review in the standard tasks of reviewers, or use specialist statistical reviewers. A significant difference between research areas was witnessed (WALD = 164.869, *df* = 13, *p* < 0.0001), suggesting stronger effects in the physical sciences and engineering as well as the life and earth sciences.

**Table 11:** Level and type of statistical review related to number of non-retracted and retracted articles in our sample

Level and type of statistical review	Retracted	95% CI	Non-retracted	95% CI
Not applicable (statistics does not play a role in my journal’s research area)	193 (28.8%)	25.4–32.2	117,760 (14.1%)	14.1–14.2
No special attention is given to statistical review	70 (10.4%)	8.2–12.7	165,637 (19.9%)	19.8–20.0
Incorporated in review (assessing statistics is part of reviewer’s and editor’s tasks)	348 (51.9%)	48.2–55.7	497,935 (59.8%)	59.7–59.9
Statistical review is performed by an additional, specialist reviewer	81 (12.1%)	9.7–14.5	152,040 (18.3%)	18.2–18.3
Statistics review is performed through automatic computer software	0 (0.0%)	0.0–0.0	4129 (0.5%)	0.5–0.5

When focussing on the different reasons for retraction, the data show that incorporated statistical review is associated with a significantly lower number of retractions due to fake review, authorship- and ethical issues (*F*(3,1303) = 63.503, *p* < 0.001). On the contrary, we do not see any substantial influence on the number of retractions due to errors or issues related to data, which arguably are more related to statistics. The effect of specialist, incorporated or IT-assisted statistics review on aspects of the manuscript directly related to data analysis remains open for further study.

The fact that retraction rates are particularly high in journals classifying statistics as ‘irrelevant’ to their research, while similar effects on retraction rates are measured for journals paying either no special attention or use specialist reviewers for statistics, would suggest that many retractions appear which are unrelated to statistics. However, additional statistics review is associated with a lower retraction rate for precisely the categories of retractions where an effect could be expected, raising additional questions. Do specialist statistics reviewers only review statistics or do they in practice consider the entire manuscript? This would, for example, explain why additional, specialist reviewers reduce the retraction rate for fake reviews. In general, higher attention for statistics is used with the intention to prevent tweaking of data or statistics. Thus it seems highly unlikely that this lower retraction rate is due to lax editorial attitudes towards problematic research. A better capability of early detection of such research seems to be a more plausible explanation even though our data cannot provide a definitive answer to this question.

### External sources

Partly due to the increasing burden on the peer review system, new procedures have emerged to reduce the number of times a single manuscript potentially needs to be reviewed through cooperation between various parties. One procedure designed to achieve this goal, is that of ‘cascading peer review’. In this procedure, (partner) journals redirect a rejected manuscript to another (potentially more suitable) journal, along with the review reports, allowing the new journal to quickly decide on the manuscript’s quality, without having to perform another round of reviews (Barroga 2013; Davis 2010). Other procedures for sharing review reports are those in which commercial review platforms assist in review (Pattinson and Prater 2017; Research Square 2017), or in which the wider community (usually in a post-publication prcedure) is invited to review a manuscript. In addition to reducing the burden on the review system, automatically (re-)directing manuscripts to the most suitable journal after review might reduce perverse incentives for authors, such as rewarding overstated conclusions to get work published. This would reduce the risk of retraction, since an incentive to overstate conclusions may provoke questionable research practices. On the other hand, it might also work in the opposite direction by relaxing review standards and allowing authors to neglect nuances, in the confidence that their work will eventually get published somewhere anyway (Horbach and Halffman 2018).

Our data (Table 12), suggest no difference in retraction rates due to the usage of review reports from external sources (*Λ*(3) = 42.270, *p* < 0.0001). No differences were observed between research areas (WALD = 1.052, *df* = 5, *p* = 0.958), nor between reasons for retraction (*F*(2, 813) = 4.166, *p* = 0.016), hence suggesting similar effects in all research areas and for all types of problematic research.

**Table 12:** Extent to which reviews from external sources are used related to number of non-retracted and retracted articles in our sample

Reviews from external sources	Retracted	95% CI	Non-retracted	95% CI
No reviews from external sources are used	422 (63.0%)	58.8–67.2	545,443 (65.5%)	65.4–65.6
Reviews from other (partner) journals are used	90 (13.4%)	10.5–16.4	244,385 (29.4%)	29.3–29.5
Reviews from commercial review platforms are used	0 (0.0%)	0.0–0.0	3971 (0.5%)	0.5–0.5
Reviews performed by the wider community are used	1 (0.1%)	0.0–0.5	3159 (0.4%)	0.4–0.4

The fact that no significant differences were found for review reports from commercial platforms or the wider community might be attributed to the low number of articles going through these kinds of review. Hence the effect of those review procedures remains to be studied. The positive effect of sharing review reports with partner journals on the number of retractions is promising, in the sense that sharing review reports potentially not only lowers the burden on the review system, but also improves the quality of the published literature. Here too, since external sources are typically used by journals trying to improve peer review, lower retraction rates are unlikely to be a sign of low willingness to act against problematic research, but rather of a high capability to detect it.

### Digital tools

One of the most promising innovations in peer review’s error and fraud detection is probably the introduction of digital tools such as plagiarism detection software, image manipulation software, software to check references (for instance for references to retracted articles), or software to assist in statistical review. Such digital tools have been implemented in a wide variety of journals with specific detection objectives (Elizondo et al. 2017; BioMed Central 2017; Scheman and Bennett 2017), and the expectation of reduced retraction rates.

Our data (Table 13) indeed suggest a significant relation between the usage of digital tools as assistance in peer review and retraction rates (Λ*(*4) = 42.270, *p* < 0.0001). In particular, more retractions occur when articles were reviewed without the assistance of digital tools and when (only) software to scan references was used. In this, subject area was a significant mediating factor (WALD = 69.496, *df* = 15, *p* < 0.0001), with stronger effects in the Social Sciences and Humanities. In addition, the usage of various digital tools has a specific effect on different reasons for retraction (*F*(4, 1880) = 27.990, *p* < 0.001). When no tools are used, we witness more retractions for plagiarism and falsification, while those retractions are sparse when plagiarism detection software is used. Similar to previous attributes, these lower retraction rates seem unlikely to be due to lax editorial attitudes towards problematic research.

**Table 13:** Usage of digital tools in peer review related to number of non-retracted and retracted articles in our sample

Level of structure		No digital tools are used	Plagiarism detection software	Digital tools to check references	Digital tools to detect image manipulation	Digital tools to assess statistics
All articles (percentage of all articles)		259,601	482,414	206,083	69,533	23,567
Non-retracted (percentage of non-retracted papers)	Total	259,321 (31.1%)	482,037 (57.9%)	205,861 (24.7%)	69,485 (8.3%)	23,561 (2.8%)
	Social Science & Humanities	25,389 (47.1%)	28,096 (52.1%)	8089 (15.0%)	5105 (9.5%)	2155 (4.0%)
	Biomedical & Health Sciences	161,264 (42.2%)	192,353 (50.3%)	50,651 (13.2%)	30,135 (7.9%)	10,622 (2.8%)
	Physical Sciences & Engineering	29,085 (14.8%)	156,573 (79.6%)	94,567 (48.1%)	7688 (3.9%)	1014 (0.5%)
	Life & Earth Sciences	36,911 (23.1%)	84,049 (51.9%)	48,009 (30.0%)	26,557 (16.6%)	8238 (5.1%)
	Mathematics & Computer Sciences	6231 (16.3%)	19,882 (52.0%)	4177 (10.9%)	0 (0.0%)	1533 (4.0%)
Retracted (percentage of retracted papers)	Total	280 (41.8%)	377 (56.3%)	222 (33.1%)	48 (7.2%)	6 (0.9%)
	Social Science & Humanities	9 (30.0%)	21 (70.0%)	7 (23.3%)	0 (0.0%)	0 (0.0%)
	Biomedical & Health sciences	242 (65.8%)	120 (32.6%)	29 (7.9%)	39 (10.6%)	6 (1.6%)
	Physical Sciences & Engineering	2 (1.1%)	180 (98.4%)	163 (89.1%)	1 (0.5%)	0 (0.0%)
	Life & Earth Sciences	26 (38.8%)	35 (52.2%)	16 (23.9%)	8 (11.9%)	0 (0.0%)
	Mathematics & Computer Sciences	1 (4.8%)	20 (95.2%)	6 (28.6%)	0 (0.0%)	0 (0.0%)

In contrast, when software to check references is used, we witness more retractions for fake review and for issues with references. The latter is clearly contrary to what should be expected, but might be explained by the sensitivity of these journals to issues with references, making them more willing to file retractions for such reasons. Here, higher retraction rates might hence be a sign of a more pro-active policy in using retractions to address issues with problematic research.

Another way of testing the effectiveness of digital tools is by comparing submissions to journals prior to and after the installation of digital tools. Because the number of changes in review procedures is relatively small, we can only meaningfully perform such an analysis specifically for plagiarism scanning tools. For this case, our results show that journals installing plagiarism software published 70,097 articles prior to the introduction of the software, leading to 38 retractions, 11 of them for plagiarism or duplication. These same journals published 41,043 articles after the introduction of the software, leading to 19 retractions, of which only 1 for plagiarism or duplication. Even though these numbers are still relatively small, it does suggest that the introduction of plagiarism software is an effective way of preventing retractions, specifically for reasons of plagiarism or duplication.

### Reader commentary

A last peer review characteristic analysed in our study concerns the extent to which journals facilitate reader commentary after the review process. Even if reader commentary is not used as a formal review mechanism, it may provide effective ways to assess manuscript quality and point out potential strengths or weaknesses to future readers. Digital technologies allow journals to provide in-channel facilities for direct reader commentary on their website, for instance in the form of blogs or forums, as well as directing readers to out-of-channel platforms that facilitate reader commentary such as *PubPeer* (PubPeer Foundation 2017). Reader commentary, and thereby heightened scrutiny on published manuscripts, may deter authors from engaging in dubious publication practices, leading to fewer retractions. At the same time, the increased detection likelihood could also increase retraction rates.

Our analyses (Table 14) demonstrate significantly higher levels of retractions with greater facilities for reader commentary, especially when in-channel reader commentary is facilitated (*Λ*(2) = 108.759, *p* < 0.0001). This suggests that higher scrutiny by readers does indeed increase the detection likelihood of problematic research reports that slipped through review, thereby leading to more retractions. In addition, we find significant differences between research fields (WALD = 20.967, *df* = 7, *p* = 0.004) and various reasons for retraction (*F*(2, 1306) = 26.607, *p* < 0.001). In particular we find strong effects in the Biomedical and Health Sciences as well as in the Life and Earth Sciences. Regarding reasons for retraction, peer review procedures with in-channel reader commentary is associated with fewer retractions due to fake review and issues with references, while there are more retractions for falsification and image/data issues compared to review procedures without direct reader commentary.

**Table 14:** Level of reader commentary related to the number of non-retracted and retracted articles per research area

Level of reader commentary		No direct reader commentary is facilitated	Reader commentary is facilitated on the journal’s website	Out-of-channel reader commentary is facilitated
All articles (percentage of all articles)		627,267 (75.3%)	203,692 (24.4%)	3735 (0.4%)
Non-retracted (percentage of non-retracted papers per research area)	Total	626,849 (75.3%)	203,441 (24.4%)	3712 (0.4%)
	Social Science & Humanities	45,185 (83.8%)	7057 (13.1%)	1691 (3.1%)
	Biomedical & Health Sciences	215,921 (56.4%)	166,460 (43.5%)	201 (0.1%)
	Physical Sciences & Engineering	185,461 (94.3%)	10,841 (5.5%)	360 (0.2%)
	Life & Earth Sciences	141,586 (88.5%)	18,473 (11.5%)	1460 (0.9%)
	Mathematics & Computer Sciences	37,781 (98.9%)	423 (1.1%)	0 (0.0%)
Retracted (percentage of retracted papers per research area)	Total	418 (62.4%)	251 (37.5%)	23 (3.4%)
	Social Science & Humanities	27 (90.0%)	3 (10.0%)	0 (0.0%)
	Biomedical & Health sciences	122 (33.2%)	246 (66.8%)	0 (0.0%)
	Physical Sciences & Engineering	182 (99.5%)	0 (0.0%)	1 (0.5%)
	Life & Earth Sciences	65 (97.0%)	2 (3.0%)	22 (32.8%)
	Mathematics & Computer Sciences	21 (100.0%)	0 (0.0%)	0 (0.0%)

The fact that more retractions appear when readers are able to comment on articles, suggests that reader commentary is a way to flag issues and put the retraction mechanism in motion. This might hence be a specific effect of how journals deal with errors in the literature. Whereas issues might be addressed internally, or in closed communication with the authors, this becomes more difficult when errors have been publicly announced and reported in reader comments. Again, higher retraction rates might hence be a sign of heightened willingness to address issues with problematic research by means of retractions. However, the extent to which reader commentary leads to retractions should be researched in more detail.

### Summary results

Combining the results from the previous sections, Table 15 presents an overview of our results. The table lists the significant correlations between retractions and peer review procedures, as well as significant interaction terms with either research area or reasons for retraction.

**Table 15:** Overview of review procedures’ significant effects on retraction rates

Review attribute	Significant effects on retractions	Significant interaction with research area	Significant variation between reasons for retraction
1. Timing	Pre-submission review is related to fewer retractions.	No	No
2. Criteria	Focussing on anticipated impact and novelty is related to more retractions.	No	No
3. Type reviewer	Involvement of editor-in-chief and wider community is related to fewer retractions.	Yes*	Yes*
4. Author anonymity	Blinding author identities is related to fewer retractions.	No	Yes
5. Reviewer anonymity	No significant effects.	No	No
6. Review reports	No significant effects in general, some effect for specific reasons for retraction and difference in effect for different research areas.	Yes*	Yes*
7. Interaction	No interaction is related to fewer retractions, having authors respond to review reports is related to more retractions.	Yes*	Yes*
8. Structure	Unstructured and structured review is related to fewer retractions, semi-structured to more.	Yes*	Yes*
9. Statistics	More retractions in journals deeming statistics ‘not relevant’, fewer retractions in journals paying no specific attention to statistics, incorporating statistics in review, or employing specialist statistics reviewers.	Yes*	Yes*
10. External sources	Using review reports from partner journals is related to fewer retractions.	No	No
11. IT-tools	Not using digital tools and using tools to check references is related to more retractions.	Yes	Yes*
12. Reader commentary	Not facilitating reader commentary is related to fewer retractions, facilitating in-channel commentary is related to more retractions.	Yes	Yes*

Significance is measured at the level of *α* = 0.01. *p< 0.001

## Limitations

Our research project may have suffered from various limitations. First, some selection and response bias may have been introduced in journal review data collection. When sampling editors’ email addresses, we searched the Web of Science for editorials written in 2017. Hence only journals indexed in the Web of Science were included. This may have caused some young, non-English, or smaller, niche journals to be excluded from our sample. In terms of publications numbers or retractions, this arguably has little effect on our general results. However, some of these excluded journals may have particularly innovative review procedures, potentially underestimating the spread of peer review innovations.

On the other hand, from the journals sampled our survey, those paying specific attention to their review process, and those particularly keen on innovating their review procedures, were arguably most likely to respond to our survey. Hence, from the journals indexed by Web of Science, we expect to have obtained responses exactly from the most innovative journals with respect to peer review procedures. Indeed, our data suggests this, with substantially more innovations reported in journals that responded very quickly to our survey request, compared to journals responding later, after one or two reminders.

To assess the effectiveness of different peer review procedures to detect fraudulent or erroneous research we used retracted journals articles as a proxy of problematic publications. We did not consider the number of published errata or corrections because these are not collected as systematically and reliably as the number of retracted articles. This approach has some limitations. First, it allows us only to trace problematic research articles that have been identified as such. Doubtless, many articles containing errors have not been retracted, either because the errors have not yet been identified, because editors are hesitant about retraction measures, authors are successfully fighting retraction measures, because of ambivalences or disagreement about what constitutes error, or a host of other reasons. Hence, our control group of non-retracted articles contains (potentially many) papers that should have been among the group of problematic articles. Nevertheless, we expect this number to be relatively small compared to the size of the control group, hence only moderately affecting our results.

Second, by using retracted journal articles, we employ problematic articles that slipped through peer review. With this method we were not able to identify problematic manuscripts that did *not* pass through peer review, i.e. those manuscripts in which errors were identified during the review process. The validity of our findings hence rests on the assumption that problematic articles were submitted via a uniform distribution to journals using different peer review models, i.e. that erroneous research was not submitted with higher probability to journals holding a specific review procedure.

A final limitation of our study rests in the survey approach to collect data about peer review procedures. Even though we tested our survey before distributing it, this type of data collection is prone to misunderstanding of the wording or confusion about definitions, as well as incomplete knowledge of the editors of (past) review procedures. This might have led editors to classify their journal’s review procedures differently from how we intended it and hence have influenced our analyses. Procedures reported by editors may not always reflect review practice.

With these limitations, we believe our research nevertheless provides valuable recommendations for journal editors to effectively design or reconsider their review procedures.

## Conclusion

In spite of various calls for more research on the effectiveness of various peer review procedures, actual evidence is rare. This study addresses precisely this knowledge gap. Our analyses reveal major differences between various ways of organising peer review and the number of retracted journal articles going through these review procedures. Thereby, they provide an indication of the effectiveness of various peer review models in detecting erroneous or fraudulent research. Even though hard causal connections cannot be made, our data suggest that some review procedures are significantly more effective in preventing retractions. In particular, author blinding seems more effective than reviewer blinding; involving the wider community in review seems beneficial; using digital tools to assist review is related to fewer retractions, as is constraining interaction between authors and reviewers, and using pre-submission review procedures such as registered reports. In addition, our data suggest differences in the effectiveness of various review procedures between scientific disciplines, as well as between specific reasons for retraction. Thereby we present a systematic comparison of review procedures’ effectiveness in detecting problematic research publications.

Our results provide specific recommendations and guidance for journal editors and publishers on how to improve the ability of their review processes to detect forms of problematic research that are particularly relevant in their subject area (with the understanding that preferences for particular peer review procedures are informed by many other considerations besides the prevention of retractions). For example, image manipulation or issues related to authorship may be of particular concern to journals in specific research areas. Our findings provide suggestions for editors to organise peer review in order to address exactly those issues, thereby allowing them to tweak review models to their specific demands.

In addition, our results suggest directions for future research in order to identify and assess the specific mechanisms underlying the effects of different review procedures. Future research should elucidate the causal connections behind the identified strong correlations. What specific mechanism, for instance, makes double-blind review better capable of detecting errors in research records than single-blind review? And what causes augmented interaction between authors and reviewers to be related to more retracted journal articles? A closer look at the practice of peer review, and particularly in those cases that led to retractions, could clarify this further. (For some very specific questions, we refer to the discussion of results per peer review procedure above.) To allow for such future initiatives, we call on journals to be more transparent about their editorial policies and review procedures. Providing such information on journals’ webpages would allow for more inclusive analyses, strengthen the power of the analyses and thereby lead to more detailed results.

Much uncertainty exists about which peer review innovations actually work. Our assessment of peer review procedures addresses this knowledge gap and may provide valuable assistance to journal editors, publishing houses, and even funding agencies. We hope this will also contribute to improved research integrity.

## Electronic supplementary material

Below is the link to the electronic supplementary material.

Supplementary material 1 (PDF 98 kb)

Supplementary material 2 (DOCX 14 kb)

## Footnotes

1 We realise this mixes theoretical expectation, data, and interpretation, but found it easier to describe what is interesting about our results than if separated in different sections.
