# Supplementary material for: The ability of different peer review procedures to flag problematic publications
Source: Scientometrics. 2018 Nov 29;118(1):339–73. doi: 10.1007/s11192-018-2969-2 (PMC6404393; doi:10.1007/s11192-018-2969-2)
Supplement: Supplementary file 1 — Supplementary material 1 (PDF 98 kb) [file 11192_2018_2969_MOESM1_ESM.pdf]

Journal information

*Required

1. **Journal name** *

2. **What is your role within this journal?**

3. **Journal's establishment date (if after January 1st 1985)**

4. **Number of submission to your journal in 2017**

5. **Number of published articles in your journal in 2017**

Timing and selectiveness

All following questions about the format of your journal's peer review allow for more than one option.

For every one of these questions, we would also like to know if the format changed since 2000. For example, you could answer: "In year X we changed review model A for review model B" or "we introduced review model A in year X". If certain review models have been used over the entire period (2000 - 2018) you do not need to specify this and may skip this question.

6. **At what stage of the publication process does review take place?** *

   Tick all that apply.

   - [ ] Post-publication review
   - [ ] Pre-publication review
   - [ ] No review takes place
   - [ ] Pre-submission review (including registered reports)

7. **Did your journal change the format of peer review since 2000 and, if so, when and how?**

8. **What quality criteria does your journal use for peer review?** *

Tick all that apply.

- [ ] Methodological rigour and correctness
- [ ] Anticipated impact (either within or outside of science)
- [ ] Novelty
- [ ] Fit with journal's scope
- [ ] Other: ____________________

9. **Did your journal change the quality criteria for peer review since 2000 and, if so, when and how?**

____
____
____
____
____

Openness of review

All following questions about the format of your journal's peer review allow for more than one option.

For every one of these questions, we would also like to know if the format changed since 2000. For example, you could answer: "In year X we changed review model A for review model B" or "we introduced review model A in year X". If certain review models have been used over the entire period (2000 - 2018) you do not need to specify this and may skip this question.

10. **What type of reviewers are included in your journal's peer review process?** *

Tick all that apply.

- [ ] Commercial review platforms
- [ ] Editor-in-chief
- [ ] Wider community / readers
- [ ] Editorial committee
- [ ] External reviewers suggested and selected by editor(s)
- [ ] External reviewers suggested by authors

11. **Did your journal change the actors involved in peer review since 2000 and, if so, when and how?**

____
____
____
____
____

12. **To what extent are authors anonimised in your journal's review process? ***
Tick all that apply.

- [ ] Author identities are blinded to editor and reviewer
- [ ] Author identities are blinded to reviewer but known to editor
- [ ] Author identities are known to editor and reviewer

13. **Did your journal change anonymity of authors in peer review since 2000 and, if so, when and how?**

14. **To what extent are reviewers anonimised in your journal's review process?**
Tick all that apply.

- [ ] Reviewers are anonymous (both to authors and other reviewers as well as to readers of the published manuscript)
- [ ] Reviewer identities are known to other reviewers of the same manuscript
- [ ] Reviewer identities are known to the authors
- [ ] Reviewer identities are known to the readers of the published manuscript

15. **Did your journal change the anonymity of reviewers since 2000 and, if so, when and how?**

16. **To what extent are review reports accessible? ***
Tick all that apply.

- [ ] Review reports are accessible to authors and editors
- [ ] Review reports are accessible to other reviewers
- [ ] Review reports are accessible to readers of the published manuscript
- [ ] Review reports are publicly accessible

17. **Did your journal change the accessibility review reports since 2000 and, if so, when and how?**

18. **To what extent does your journal's review process allow for interaction between reviewers and authors?** *
Tick all that apply.

- [ ] No interaction between authors or reviewers is facilitated
- [ ] Interaction between reviewers is facilitated
- [ ] Author's responses to review reports are communicated to the reviewer
- [ ] Interaction between authors and reviewers is facilitated (on top of formal review reports and formal responses to review reports)

19. **Did your journal change the extent of interaction during peer review since 2000 and, if so, when and how?**

Specialisation of review

All following questions about the format of your journal's peer review allow for more than one option.

For every one of these questions, we would also like to know if the format changed since 2000. For example, you could answer: "In year X we changed review model A for review model B" or "we introduced review model A in year X". If certain review models have been used over the entire period (2000 - 2018) you do not need to specify this and may skip this question.

20. **To what extent is your journal's review process structured?** *
Tick all that apply.

- [ ] Structured: Review occurs through mandatory forms or checklists to be filled out by reviewers
- [ ] Unstructured: reviewers are free to choose how to organise their review and are not presented questions or criteria for judgement
- [ ] Semi-structured: Reviewers are guided by some (open) questions or are presented several criteria for judgement

21. **Did your journal change the structure of peer review since 2000 and, if so, when and how?**

22. **To what extent does your journal's review process use specialist statistical review?** *
Tick all that apply.

- [ ] Not applicable (statistics does not play a role in my journal's research area)
- [ ] No special attention is given to statistical review
- [ ] Incorporated in review (assessing statistics is part of reviewer's and editor's tasks)
- [ ] Statistical review is performed by an additional, specialist reviewer
- [ ] Statistics review is performed through automatic computer software

23. **Did your journal change the format for statistical review since 2000 and, if so, when and how?**

24. **To what extent does your journal accept or use reviews from external sources?**
Tick all that apply.

- [ ] No reviews from external sources are used
- [ ] Reviews from other (partner) journals accompanying manuscripts rejected elsewhere are used
- [ ] Reviews from commercial review platforms are used
- [ ] Reviews performed by the wider community (i.e. not by invited or targeted reviewers) are used (e.g. reviews on public forums)
- [ ] Other: ____________

25. **Did your journal change the extent to which external reviews are used since 2000 and, if so, when and how?**

Technological support in review

All following questions about the format of your journal's peer review allow for more than one option.

For every one of these questions, we would also like to know if the format changed since 2000. For example, you could answer: "In year X we changed review model A for review model B" or "we introduced review model A in year X". If certain review models have been used over the entire period (2000 - 2018) you do not need to specify this and may skip this question.

26. **What forms of digital tools are used in your journal's review process?** *
Tick all that apply.

- [ ] No digital tools are used
- [ ] Digital tools to check references are used (e.g. to check for references to retracted articles, or references to articles published in predatory journals)
- [ ] Plagiarism detection software is used
- [ ] Digital tools to assess validity or consistency of statistics are used
- [ ] Digital tools to detect image manipulation are used
- [ ] Other: ____________________

27. **Did your journal change the use of digital tools in peer review since 2000 and, if so, when and how?**

____
____
____
____
____

28. **To what extent does your journal's review process allow for reader commentary after publication of a manuscript?** *
Tick all that apply.

- [ ] No direct reader commentary is facilitated
- [ ] Reader commentary is facilitated on the journal's website
- [ ] Out-of-channel reader commentary is facilitated (e.g. by providing links to commentary on other platforms such as PubPeer)

29. **Did your journal change the format for reader commentary since 2000 and, if so, when and how?**

____
____
____
____
____

Results

30. **Our project aims to contribute to the evidence-base regarding the effectiveness of various peer review formats in detecting erroneous and fraudulent research. In case you would like to be updated on our project's results, please leave your email address in the box below.**

Powered by
Google Forms
