# Supplementary material for: The ability of different peer review procedures to flag problematic publications
Source: Scientometrics. 2018 Nov 29;118(1):339–73. doi: 10.1007/s11192-018-2969-2 (PMC6404393; doi:10.1007/s11192-018-2969-2)
Supplement: Supplementary file 2 — Supplementary material 2 (DOCX 14 kb) [file 11192_2018_2969_MOESM2_ESM.docx]

**Categorisation of reasons for retraction**

| Reason for Retraction | Retraction Watch categories |
| --- | --- |
| plagiarism / duplication of text | Duplication of Article  Duplication of Text  Euphemisms for Duplication  Euphemisms for Plagiarism  Plagiarism of article  Plagiarism of Text |
| Error | Error in Analyses  Error in Cell Lines/Tissues  Error in Data  Error in Image  Error in Materials (General)  Error in Methods  Error in Results  Error in Text  Results Not Reproducible  Sabotage of Materials |
| Falsification / Manipulation | Manipulation of Results  Manipulation of Images  Falsification/Fabrication of Results  Falsification/Fabrication of Image  Falsification/Fabrication of Data |
| Image / Data Issues | Concerns/issues about data  Concerns/issues about image  Concerns/issues about results  Duplication of data  Duplication of image  Manipulation of Images  Plagiarism of Image  Plagiarism of Data  Unreliable Data  Unreliable Image |
| Fake Review | Fake Peer Review |
| Authorship issues | Forged Authorship  Conflict of interest  Concerns/issues about authorship |
| Ethical violations | Lack of IRB/IACUC Approval  Ethical Violations by Author  Conflict of interest |
| Issues with references | Concerns/issues about referencing  Cites prior retracted work |
| Misconduct | Salami Slicing  Sabotage of Materials  Plagiarism of Image  Plagiarism of Text  Plagiarism of Data  Plagiarism of Article  Misconduct by Third Party  Misconduct by Company/Institution  Misconduct by Author  Misconduct - Official Investigation/Finding  Manipulation of Results  Manipulation of Images  Informed/Patient Consent - None/Withdrawn  Forged Authorship  Falsification/Fabrication of Results  Falsification/Fabrication of Image  Falsification/Fabrication of Data  Fake Peer Review  Euphemisms for Plagiarism  Euphemisms for Misconduct  Euphemisms for Duplication  Duplication of text  Duplication of image  Duplication of data  Duplication of article  Conflict of interest |
